# Understanding the Utilization of Wasted Bread as a Brewing Adjunct for Producing a Sustainable Wheat Craft Beer

**DOI:** 10.3390/microorganisms13010066

**Published:** 2025-01-02

**Authors:** Katry Dall’Acua, Manuela Poletto Klein, Bárbara Iegli Tech, Alessandra Fontana, Ludmylla Tamara Crepalde, Roger Wagner, Fernanda de Candido de Oliveira, Voltaire Sant’Anna

**Affiliations:** 1 Life and Environmental Area, State University of Rio Grande do Sul, Encantado 95960-000, Brazil; katry-acua@uergs.edu.br (K.D.); manuelap@ufcspa.edu.br (M.P.K.); barbara-tech@uergs.edu.br (B.I.T.); voltaire-santanna@uergs.edu.br (V.S.); 2 Nutrition Department, Federal University of Health Sciences of Porto Alegre, Porto Alegre 90050-170, Brazil; 3 Department for Sustainable Food Process (DiSTAS), Faculty of Agriculture, Food and Environmental Sciences, Catholic University of the Sacred Heart, 26100 Cremona, Italy; 4 Department of Food Technology, Federal University of Viçosa (UFV), Viçosa 36570-900, Brazil; ludcrepalde@gmail.com; 5 Department of Food Science and Technology, Federal University of Santa Maria, Camobi, Santa Maria 97105-900, Brazil; rogerwag@gmail.com (R.W.); fernandac1@icloud.com (F.d.C.d.O.)

**Keywords:** ale beer, wasted bread, food waste, sugars, volatile compounds, sensory analysis

## Abstract

Wasted bread (WB) has been studied as an alternative ingredient for increasing the sustainable footprint in the beer production chain. There are gaps in the literature on the impact of WB on beer manufacturing. Thus, the objective was to evaluate the addition of WB as a replacement for wheat flakes in a craft beer. Three formulations with different concentrations of WB were produced and monitored for glucose and maltose concentrations in the mash; the beer was analyzed for ethanol, glycerol, acetic acid, lactic acid, pH, acidity, turbidity, color, and volatile compounds. Sensory analysis was performed by a trained panel. In the initial stages of mashing, a higher concentration of sugars was found in the wort with WB added, while, at the end stages, this was higher in the control wort. The addition of WB resulted in beers with a lower turbidity, darker color, and lower concentrations of ethanol, glycerol, and acetic acid. Among the volatile compounds, D-limonene, ethyl dodecanoate, heptanol, acetaldehyde, and ethyl acetate should be further explored as markers for the presence of WB. Higher intensities of banana odor and flavors were observed by the trained panel when there was a greater substitution of wheat flakes. WB is a low-cost and effective ingredient for beer production, although more work is needed for its large-scale use.

## 1. Introduction

Bread is one of the most popular bakery products worldwide, but it has been reported to be highly wasted at the household level due to a complex multifactorial process that involves physical staling and microbiological, chemical, and sensorial spoilage, limiting its shelf-life [1,2]. About 17% of foods are globally wasted currently; thus, avoiding food waste is a major challenge, and this is highlighted in target 12.3 of the Sustainable Development Goals [3]. Eriksson et al. [1] observed that the donation of bread that would be wasted is a better option for their destination when compared with landfill disposal, composting, and aerobic digestion with regard to greenhouse gas emissions, mainly considering the amount of CO_2_/kg generated and the nutrient recovery aspects. It is difficult to quantify the precise amount of wasted bread (WB); annual global bread production exceeds 100 million tons, and the estimated waste for bakery foods is around 10 million tons worldwide [4,5]. Bread loss does not simply reflect the loss of the product itself but also the loss of various natural resources, such as the water, land, and energy used for the production of raw materials, transportation, and manufacturing [5].

Upcycled foods are defined as products consisting of or containing materials that otherwise would be wasted; these materials are turned into food products for human consumption, and this is carried out via a process that involves an increase in value [6]. The utilization of bread that has gone stale has been cited as a potential ingredient in producing upcycled foods [6]. In this scenario, the implementation of circular economy concepts along the food chain is an essential step in order to avoid wasting food ingredients that present valuable properties for food applications, thus achieving the Sustainable Development Goals [6]. Wasted bread is an important source of carbohydrates (expressed as starch) and proteins that can be employed in the fermentation process to avoid the waste of energetic compounds. It has been proposed for use as a substrate for enzyme production, yeast biomass, organic acids, and beer, among others [7,8].

Beer is one of the most popular beverages worldwide, where carbohydrates from malted barley and/or non-malted adjuncts (corn, oat, and wheat) are used as carbon sources for yeast fermentation to generate alcohol and compounds of flavor and texture [7]. In this context, starch from bread is a source of easily fermentable sugars that results from the action of α- and β-amylases in malt during beer mashing [9,10]. Therefore, it is interesting to concentrate on waste streams with a high sugar content, where the extraction of fermentable sugars is convenient and low-cost, such as food, bakery, bread, fruit, and beverage waste, and these have received significant attention in recent years [11]. Wheat beer can contain a maximum content of 80% by weight of all beer adjuncts in relation to its original extract and a minimum of 20% by weight of barley malt [12]. Therefore, WB fits as an adjunct, and, in the formulations tested, it did not reach the maximum content permitted by law.

In Brazil, craft beer follows ordinary legal standards for ingredients and processing facilities [12], and craft breweries differ from large companies mainly in terms of the volume of beverage produced. The relatively high selling price of craft beers has become an obstacle to market expansion and increased consumption [13]. Additionally, Diniz and Carvalho [14] observed that the acquisition of raw materials for craft beers has an important role in the generation of greenhouse gas emissions by this sector; therefore, the utilization of wasted bread as a brewery adjunct is a strategy for reducing ingredient costs, avoiding food waste, and shortening the ingredient purchasing chain for microbreweries.

Martin-Lobera et al. [9] recently studied a pale ale beer with several kinds of wasted bread (wheat, rye, corn, and brown wheat) in a single addition of this byproduct into the brewery protocol and observed that beers are important vehicles for the valorization of this residue, since the beverage yields quite similar physicochemical and sensorial performances. However, exploring the effect of wasted bread on other beer types and formulations is essential to its proper insertion into the industrial routine. Additionally, understanding how wasted bread influences the wort during the brewing process has not been explored, which is critical data for standardizing beer production. Within these contexts, the objective of the present work was to evaluate the substitution of barley malt for wasted bread in ale beer and to evaluate the impact on the beverage during the brewing step and in the final product. Based on this, the conversion of WB starch into fermentable sugars was assessed, along with an evaluation of the physicochemical properties, volatile compounds, and sensory profile of the final product.

## 2. Materials and Methods

### 2.1. Ingredients and Chemicals

French-style bread from a local bakery (containing wheat flour, eggs, soy oil, salt, sugar, and bakery yeast; data about the quantities and bake conditions were not provided) was purchased one day before their shelf-life expiration (three days after production). The bread was cut into pieces, dried in an industrial oven (SPLabor, SP 102/27 model, Encantado, Brazil) at 200 °C for 20 min, cooled to room temperature, and milled in a domestic blender. The malt, hops, and yeast were kindly provided by Cervejaria Imigração (Campo Bom, Brazil).

Chemicals for high-performance liquid chromatography (HPLC) were obtained from Sigma Aldrich (St. Louis, MO, USA) and other chemicals from Êxodo Científica (Sumaré, São Paulo, Brazil).

### 2.2. Beer Production

In the present work, an ale type of craft beer was produced. Brewing was performed in 10 L lab-scale pans, by using four formulations (Table 1).

Brewing was carried out following the protocol proposed by Díaz et al. [15] with few modifications. In brief, the wheat flakes were mixed with 10 L of mineral water at 45 °C for 15 min. After adding the malts and bread, the temperature was raised to 50 °C at a rate of approximately 1 °C per minute and was then held for 15 min. Afterwards, the temperature was increased by 1 °C per minute until reaching 65 °C, where it was maintained for 60 min. Finally, the temperature was raised at the same rate to 75 °C and held for 15 min. The system was filtered using a malt bagasse, and 4 L of mineral water at 76 °C were added to wash the bagasse. Afterward, it was boiled for 60 min, and, in the final 30 min, hops (Herkules) were added and cooled down to room temperature, when yeast Heiffeweizen (Bio4—http://bio4.com.br/produtos/heffeweizen-ale/, accessed on 10 November 2024) (*Saccharomyces cerevisiae*) was added. Fermentation occurred at 18 °C in a temperature-controlled chamber for 10 days. The resulting beer was then bottled in 500 mL bottles and 6 g/L of commercial sugar was added as priming agent. The final product was left to stabilize at 25 °C for 10 days, and then refrigerated. Samples from each brewing stage were withdrawn and frozen at −18 °C until analysis. Before analysis, samples were thawed overnight at 5 °C and degassed in an ultrasonic bath (Unique^®^, USC-800, Indaiatuba, SP, Brazil) for 5 min.

### 2.3. pH, Acidity, Turbidity, and Color Measurements

The pH analysis was performed using a benchtop digital pH meter (PHOX P1000, PHOX Suprimentos Científicos, Colombo, PR, Brazil). The total acidity was determined by titration with standardized 0.01 N sodium, and results were expressed as percentages [16]. Turbidity was measured with a digital turbidimeter (DLT-WV, DEL LAB, Araraquara, SP, Brazil) and expressed as nephelometric turbidity units (NTUs). Color analyses were performed measuring the absorbance at 430 nm using a UV–Vis spectrophotometer (IL-226, Kasuaki, Tokyo, Japan), and results were converted in the European Brewery Convention (EBC) scale, multiplying the value by 25 [9]. Moreover, the CIELab (L*, a*, b*) parameters were measured by determining the D-65 diffuse illumination and with a 10° observer of a colorimeter (Konica Minolta Chroma CR-400, Tokyo, Japan). Chroma (C*) was calculated by Equation (1) and the difference of color (Δ*E*) by Equation (2) [17]:

(1) $$C^{*}=\sqrt{a^{*2}+b^{*2}}$$

(2) $$\Delta E=\sqrt{{\left(L_{control}^{*}-L_{bread\;beer}^{*}\right)}^{2}+{\left(a_{control}^{*}-a_{bread\;beer}^{*}\right)}^{2}+{\left(b_{control}^{*}-b_{bread\;beer}^{*}\right)}^{2}}$$

### 2.4. Ethanol, Acetic Acid, Lactic Acid, Glycerol, Glucose, and Maltose Analyses

Ethanol, acetic acid, lactic acid, and glycerol, as well as glucose and maltose concentrations, were determined by an HPLC system (Shimadzu, Kyoto, Japan) equipped with a refractive index detector and a Bio-Rad HPX-87H column (300 × 7.8 mm) using 5 mM sulfuric acid as eluent at 45 °C and flow rate of 0.6 mL/min [18,19]. Samples were centrifugated at 10,000× *g* and filtered (0.45 μm membrane) before the injection of 20 μL aliquots. Standards of the analyzed compounds were used for identification (retention time) and quantification (external standard). The results were expressed as mg/L.

### 2.5. Volatile Compound Profile

The volatile organic compounds (VOCs) of beer samples were analyzed using the solid-phase microextraction technique applied to the sample headspace (HS-SPME). A divinylbenzene/carboxen/polydimethylsiloxane (DVB/Car/PDMS; 2 cm × 50/30 µm, Supelco, Bellefonte, PA, USA) sorbent fiber was used in the analysis. The extraction procedure was carried out according to [19] with modifications. Briefly, 5 mL of beer was added in a 20 mL glass vial, with1.5 g of NaCl and 100 μL of a 3-octanol internal standard solution (10.8 mg/L). VOCs were extracted at 35 °C for 60 min, with a previous 10 min of equilibrium time of the temperature without fiber exposition. The fiber was conditioned before the analysis as recommended by the manufacturer.

The VOCs were then analyzed using a gas chromatograph coupled to a mass spectrometer (GC/MS) (QP-2010Plus, Shimadzu, Kyoto, Japan). After the extraction, the SPME fiber was desorbed into the GC injector port at 250 °C, in splitless mode (1 min split-valve off, followed by a split 1:5). The analytes were separated on a ZB-Wax capillary column (60 m × 0.25 mm; 0.25 μm stationary phase thickness; Phenomenex, Torrance, CA, USA). The oven temperature was initially set at 35 °C for 5 min, then increased at a rate of 5 °C min^−1^ until 230 °C, and held for 5 min. The GC/MS interface and ionization source were operated at 230 and 200 °C, respectively. The mass spectrometer was set to electron ionization mode, and the mass analyzer scanned between 35 to 350 *m*/*z*. Compounds positively identified by comparing the retention times and spectra of standard compounds and those identified by comparing the experimental mass spectrum with those found in the NIST 14 spectra library are found in the Section 3. Additionally, the experimental linear retention index of each compound (calculated by a series of n-alkanes under the same sample analysis conditions) was compared with those found in the literature for columns with similar stationary phases. The 3-octanol was used as an internal standard to semi-quantify the compounds, and the results were reported in µg/L.

### 2.6. Descriptive Analysis (DA)

Sensory analysis was carried out in the Central Location Test (individual tables in a neutral room (20 ± 2 °C) in Campo Bom (Brazil). The panel consisted of 10 brewery experts, both male and female, with extensive experience in tasting craft beers. The descriptive analysis (DA) methodology was used, evaluating four appearances (yellow color, dark color, turbidity, and foaming), four olfactory (banana, clove, malt, and floral aromas), nine gustatory (banana, clove, malt, alcoholic tastes, acidity, sweetness, refreshment, bitterness, and sparkling), and two aftertastes (persistent taste in the mouth and residual taste) items, based on an internal quality control report from a local brewery and literature data [20,21]. The description of attributes is found in Supplementary Material (Table S1). Attributes intensities were rated based on a 9 cm non-structured scale anchored on “none/weak” and “very strong”. The samples were randomly served to the judges in beer glasses at a temperature of 10 °C, accompanied by mineral water for palate cleansing between tastings.

Prior to participating in the study, individuals were provided with an information sheet detailing all aspects of the study and were required to give written consent. Each participant was assigned a unique code to maintain anonymity. No financial incentive was provided and the project was conducted in agreement with the Declaration of Helsinki and approved by the Human Research Ethics Committee (protocol number: 80964624.8.0000.8091).

### 2.7. Statistical Analysis

Analyses were performed using R studio (version 2023.12.1) and XLSTAT (version 2023.2.0) (Lumivero, New York, NY, USA). The α-risk was set at 0.05. The means of three replicates from physicochemical parameters were compared by one-way analysis of variance (ANOVA), followed by Tukey’s HSD test. The normality of the data and homogeneity of the variances were tested before ANOVA by Shapiro–Wilk and Hartley’s maximum F tests, respectively, at Rstudio software (version 2024.12.0).

DA sensory data was analyzed in PanelCheck software (PanelCheck software, 2006, http://www.panelcheck.com, accessed on 20 August 2024) to evaluate the significance of the interaction of panelist*samples, which was considered satisfactory when *p* > 0.05 using ANOVA, followed by Least Squared Difference (LSD) test. Duplicate means were compared using two-way ANOVA, with samples treated as a fixed effect and consumers as a random effect, followed by Tukey’s test.

For volatiles, means were centered and scaled for variance one in a column-wise direction to prevent the differences in the scale of volatile metabolite abundances from affecting the assessment of sample differences. The heatmap was built using pheatmap package (version 1.0.12) [22] in R (version 4.0.3).

Multiple Factor Analysis (MFA) was performed using sample triplicates at software XLSAT (Addinsoft, Paris, France, version 2021.3.1).

## 3. Results

### 3.1. Physico-Chemical Features During Brewing and Beers

**Figure 1:**
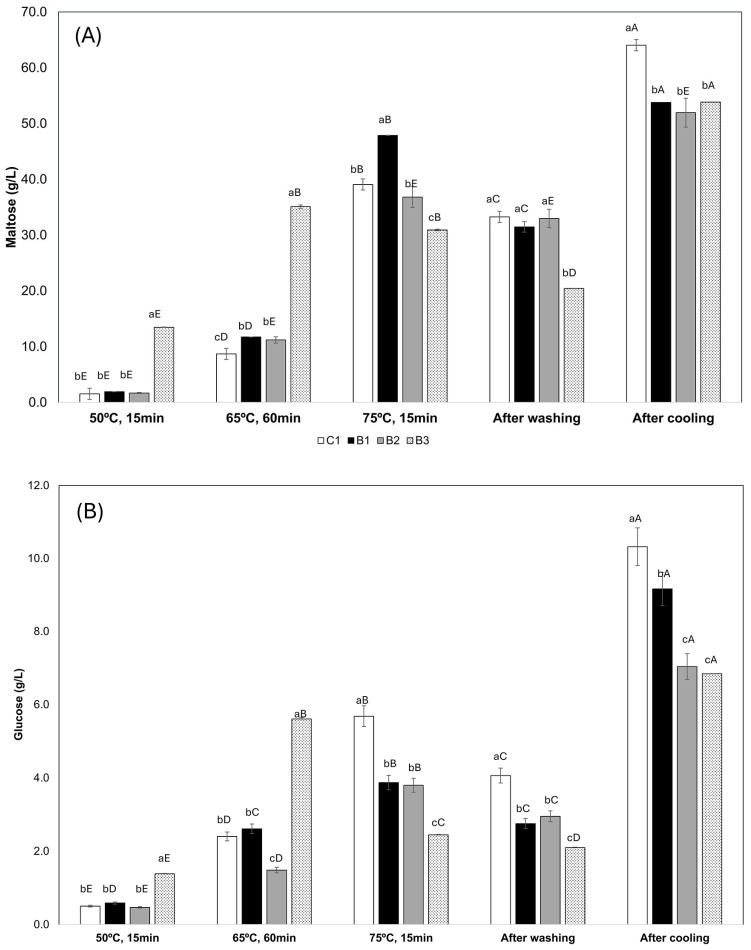
Maltose (**A**) and glucose (**B**) changes on wort during beer brewing added with WB. In the white column—control; in the black column—BB1; in the gray column—BB2; and in the white column with dots—BB3. Through analysis of variance, followed by Tukey’s HSD test, a, b, c, different lowercase letters, indicate statistical differences among treatments in the same brewing process at 5% of significance. A, B, C, D, E, different capital letters, indicate statistical differences among brewing process in the same treatment at 5% of significance.

The changes in maltose and glucose content in beer wort during the brewing process are shown in Figure 1.

In the first mashing ramp, the concentration of maltose (Figure 1A) and glucose (Figure 1B) did not differ (*p* > 0.05) between the control and samples where wheat flakes were partially substituted by WB (BB1 and BB2). Only the samples where WB completely replaced wheat flakes (BB3) showed a higher concentration (*p* < 0.05) for both the sugars. In the second ramp, sugars increased for all samples; however, samples added with WB presented a significantly higher concentration of maltose than the control. In contrast, for the glucose concentration, the control sample did not differ significantly (*p* > 0.05) from BB1, which was higher than BB2. Wort with a complete substitution of wheat flakes maintained higher amounts of both glucose and maltose. The maltose concentration kept increasing from ramps 2 and 3 in the control samples and those with a partial substitution of wheat flakes (BB1 and BB2), but BB3 (with a complete substitution of wheat flakes for WB) did not change. In ramp 3, instead, a higher amount of maltose was observed in BB1 (Figure 1A); meanwhile, higher glucose was observed in the control sample (Figure 1B). After the washing process, the amount of both sugars was reduced and the maltose concentration was higher in the control, BB1, and BB2 samples; meanwhile, higher concentrations of glucose were observed in the control only. BB1 and BB2 did not differ for both sugars, whereas BB3 presented a lower concentration. At the end of the brewing process, the results showed that control samples presented higher concentrations of maltose and glucose (*p* < 0.05) than samples with WB. Regarding maltose, the wort containing WB did not differ (*p* > 0.05) from the control, whereas BB2 presented the highest concentration of glucose among WB-added beers.

Some characteristics of the produced beers are presented in Table 2. The pH of the control beer differed significantly (*p* < 0.05) from all the other samples. Instead, BB1 and BB2 did not show any difference (*p* > 0.05) and the acidity did not differ between all samples (*p* > 0.05), with pH and acidity values close to 4.0 and 0.25%, respectively. For the appearance parameters, the results showed that beer with only wheat flakes (control) presented the highest turbidity, whereas beer with 70% substitution of wheat flakes for wasted bread (BB2) had the lowest turbidity. The highest EBC values, indicating a darker color, were observed in the control samples, while the lowest values were found in BB1 and BB3. The opposite trend was observed for the lightness (L*) values, which were higher for BB1 and BB3 samples than the control. Reddish color (a*-values) and opacity (C*-values) did not differ among samples with wasted bread (*p* > 0.05); meanwhile, yellowish color (b*-values) did not differ among all samples (*p* > 0.05). The highest difference in color (ΔE) was observed in the BB1 sample, whereas the lowest was in BB2.

The ethanol concentration did not differ between the control and the WB-partially-substituted beers, whereas beers with WB only as the wheat source presented the lowest ethanol content. The glycerol concentration did not differ among samples (*p* > 0.05); instead, considering acetic acid, the highest and the lowest concentrations were observed in the control and totally WB-substituted samples, respectively. In contrast, a significantly higher concentration of lactic acid was observed in BB3 than the other samples. Regarding the glucose and maltose content, the first did not differ between samples; meanwhile, maltose was not found in the control samples, and, between wasted bread beers, its content did not differ significantly.

### 3.2. VOC Profile of Beers

Regarding VOC, Table 3 shows the 45 volatile compounds identified among samples, which were classified as acids (4), alcohols (12), aldehyde (1), esters (21), ketones (2), terpenes (4), and polycyclic aromatic hydrocarbon (1). According to the Beer Judge Certification Program [23], wheat beer is characterized by the presence of moderate to strong esters and phenols, typically banana and clove, generally well-balanced, with a light to moderate aroma reminiscent of bread, bread dough, and/or cereals such as wheat, and lightly floral, spicy, and/or herbal hops.

Among volatile acids, hexanoic, 5-methylhexanoic, heptanoic, and octanoic acids were identified. Higher amounts of all acids were observed in the control sample (*p* > 0.05), except for 5-methylhexanoic acid, which did not differ (*p* > 0.05) from the beer with the lowest substitution of wheat flakes for wasted bread (BB1). Among the wasted bread beers, for octanoic acid, which presents a recognized sweat/cheesy odor [24,25], a higher amount was observed in BB1 (*p* < 0.05), followed by BB2 and BB3, which did not differ between them (*p* > 0.05). For volatile alcohols, overall, higher amounts were observed in the control samples, and a higher addition of wasted bread led to a reduction in the alcohols’ presence. Hexanol (flower/green odor) [24,25], propanol (alcohol/pungent odor) [25], butanol (medicine/fruit odor) [25], 3-methyl butanol (whiskey/malt odor) [25,26], and phenethyl alcohol were in a higher (*p* < 0.05) amount in the control samples; meanwhile, the amount of 2-methyl butanol (malt odor) [25] and octanol (chemical/metal/burnt odor) [26] did not differ among samples (*p* > 0.05). The only aldehyde identified as acetaldehyde, which presented green/apple notes [27], showed the highest value in control samples (*p* < 0.05) and did not differ among the wasted bread beers (*p* > 0.05). Twenty-one esters were identified within the samples.

**Figure 2:**
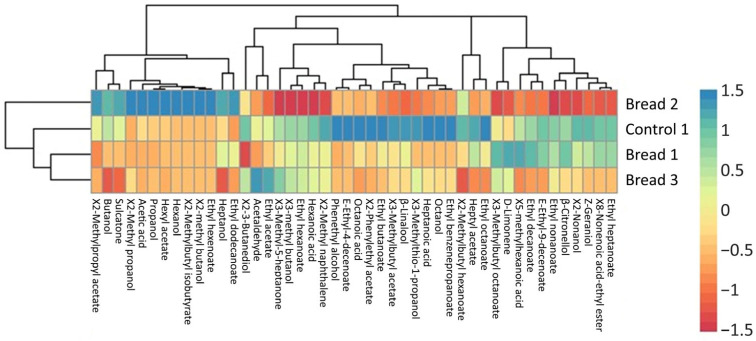
Heatmap representing the VOCs’ profile of the different beer samples (control, BB1, BB2, and BB3).

Based on the literature, recognized odors due to the presence of ethyl acetate (pineapple odor), ethyl hexanoate (fruit/jelly palm) [28], ethyl octanoate (fruit odor) [25], and hexyl acetate (fruit/herb odor) [25] showed the highest levels in control samples (*p* < 0.05), whereas wasted bread beers did not present significant differences (*p* > 0.05). Among ketones, a higher amount of 3-methyl-5-heptanone was observed in BB2; meanwhile, 6-methyl-5-hepten-2-one was in a higher amount in the control samples (*p* < 0.05). Considering terpenes, D-limonene, with a recognized lemon/orange odor [29], showed the highest and lowest concentration in BB1 and BB3 samples, respectively; instead, β-linalool (flower/lavender odor) [30], β-citronellol, and Z-geraniol (both with rose odors) [26] presented higher amounts in control samples. VOCs’ distribution between the investigated samples is shown in Figure 2. 

In control samples, a dominance of octanol, 3-(methylthio)-1-propanol, heptanoic and octanoic acids, 3-methylbutyl acetate, ethyl butanoate, phenylethyl acetate, ethyl octanoate, ethyl decanoate, ethyl benzenepropanoate, and linalool was evidenced. On the other hand, BB2 mostly included ethyl hexenoate, 2-methyl butanol, hexanol, 2-methyl propanol, propanol, acetic acid, 2-methyl isobutyrate, hexyl acetate, and 2-methylpropyl acetate, with a lower presence of hexanoic acid, 3-methyl butanol, ethyl hexanoate, ethyl heptanoate, ethyl 8-nonenoate, Z-geraniol, β-Citronellol, D-Limonene, and 2-methyl naphthalene. BB3 was marked instead by a lower presence of butanol, heptanol, sulcanote, and 2-methylbutyl hexanoate.

### 3.3. Beers’ Sensory Profile

**Figure 3:**
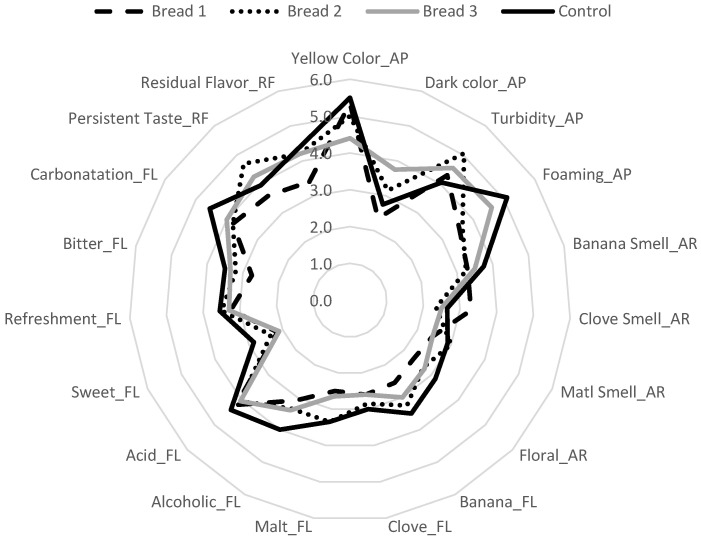
Intensity average values of the sensory attributes evaluated in the Descriptive Analysis. The letters after the attributes stand for the following: AP—appearance; AR—aroma; FL—flavor; and RF—residual flavor.

The sensory profiles of the investigated beers are shown in Figure 3. For appearance attributes, the highest dark color (*p* < 0.05) was observed in bread beer with a total substitution of wheat flakes for wasted bread (BB3), whereas the other samples did not differ between them (*p* > 0.05). Regarding foam formation, the control and BB3 samples did not show a significant difference and presented the highest intensity (*p* < 0.05), while beers with a partial substitution of wheat flakes did not significantly differ between them. Considering the aroma attributes, the banana smell had higher scores in the control and BB3 beers (*p* < 0.05); meanwhile, BB1 and BB2 did not differ; for the clove, malt, and floral aroma, all samples did not differ in terms of their intensities (*p* > 0.05).

With regard to flavors, control, BB2, and BB3 presented the highest intensity of banana flavor. Instead, for the malt flavor, only control and BB2 presented the highest intensity (*p* < 0.05). Control samples also showed the highest alcoholic flavor and carbonatation compared to the wasted bread beers (*p* < 0.05). There were no significant differences between the samples for clove, acidic, sweet, refreshing, and bitter taste. Considering the after-taste attributes, BB2 showed the highest intensity in the persistent flavor, while the residual flavor did not significantly differ between samples. The total replacement of wheat flakes with WB was not perceived by the trained panel.

**Figure 4:**
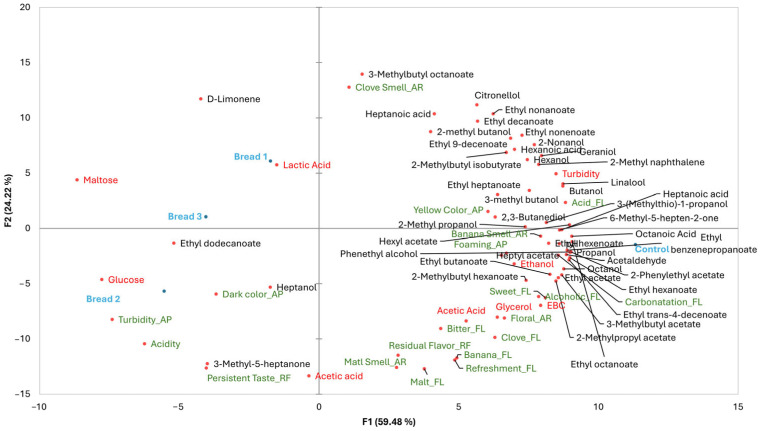
MFA biplot including all the parameters investigated in the beers under study. Blue, red, black, and green colors represent the samples, physico-chemical parameters, VOCs, and sensory attributes, respectively.

The MFA, including all the parameters investigated in the study (Figure 4), explained, on the two axes, 83.70% of variance.

It is shown that wasted bread beers are clustered together, and they were separated, on F1, from the control beer. The MFA also evidenced that D-limonene, ethyl dodecanoate, and heptanol might represent volatile markers of the presence of wasted bread in the beer samples, as well as the turbidity, acidity, dark color, and persistent taste. Instead, ale beers without bread residues were mostly marked by a banana aroma, sweet flavor, and a wide range of VOCs, indicating a greater bouquet of flavors/aroma.

## 4. Discussion

The brewing process aims at hydrolyzing starch malt and non-malted ingredients into fermentable sugars (i.e., maltose—a disaccharide—and glucose—monosaccharide). During the mashing steps, an increase in maltose and glucose, due to the action of amylase enzymes in the wort, was evidenced for all the samples. It is worth noting that the amount of malted barley in the beers was kept consistent to ensure the same diastatic activity, unlike other studies where malt was substituted with non-malted adjuncts. It was shown that, during the first two brewing steps, a complete substitution of wheat flakes for wasted bread resulted in a higher release of maltose and glucose in the wort. This can be mainly due to the presence of available sugars in bread. During the first brewing step (50 °C for 15 min), there is mainly the action of proteolytic enzymes that break down the protein chain, producing peptides and amino acids [31], and the action of the enzyme limits dextrinase, which breaks down the α-1,6 bonds of amylopectin, favoring the action of β-amylase [32,33]. In the second temperature ramp (65 °C for 60 min), β-amylases become more intense, acting on the α-1,4 bonds at the end of poly/oligosaccharide chains [34], releasing maltose. In the third stage (76 °C for 15 min), there is mainly the action of α-amylases on the α-1,4 bonds between glucose, commonly in the middle of the starch chain, and can generate glucose, maltotriose, maltose, and dextrins [34]. These phenomena help to explain the high increase in both sugars in samples with wheat flakes (control, BB1, and BB2) and the constant amount in BB3.

The reduction in sugar concentrations after the washing stage and the increase after the boiling/cooling stage are related to the addition of water in the first stage and the evaporation of water in the second stage, respectively.

In the cooled wort, after the boiling process, the concentration of maltose was higher in the control formulation, which did not contain wasted bread. It was noted that the maltose levels did not present a significant difference in the formulations that contained bread. The glucose concentration was higher in the control formulation, but the beer with the lowest amount of bread (BB1) had a higher glucose content than the formulations BB2 and BB3. A relevant factor is that the wort takes a long time to reach the boiling point, and the action of the enzymes can continue to slowly hydrolyze the starch since important diastatic power in the wort remains at temperatures over 72 °C [35]. It is noted that the beer samples with wasted bread released a smaller amount of sugar, which may have occurred due to the interference of some non-starchy substances, products of the Maillard reaction, and the caramelization of sugars, or the presence of proteins and lipids on the surface of the starch granules, reducing and interfering with the hydrolysis of the starch in the bread [36]. Wasted bread is a bakery product that has undergone a starch gelatinization process—the breaking of starch granules, in the presence of water and high temperature. After the bread goes through the cooling process and the starch is already gelatinized, the retrogradation process begins. This process begins with the recrystallization of amylose and then with amylopectin [37]. Retrogradation leads to the formation of crystalline structures and a lower water content, which would be an obstacle to hydrolysis by amylases [36], favoring a greater formation of non-fermentable sugars and, consequently, resulting in a lower release of fermentable sugars at the end of the mashing process.

Higher concentrations of maltose and glucose were observed in the wort before fermentation. In the finished beers, the concentration of maltose and glucose decreased during the fermentation process. In beers with the addition of bread, maltose was present, and, in all samples, glucose was present. It is assumed that residual sugar was formed or that the yeast did not consume all the added sugar during carbonation and the addition of sugar to the bottles.

There were no significant differences in the ethanol concentration between WB beers and the glycerol concentration was not affected by the presence of bread in all beers. The production of glycerol, a secondary metabolite of ethanol, by the yeast, was less than 1 g/L and did not imply improvements in the body of the beverages or in their flavor [38].

In the produced beers, there was the presence of lactic acid and acetic acid in all formulations. In most cases, the presence of noticeable lactic acid is considered an off-flavor; however, bacteria that can produce this acid are generally considered beer contaminants, although there are cases where they are desired [39]. Acetic acid can be produced by yeasts as a natural byproduct, but it can also be produced by aerobic bacteria genera that, under excessive aeration conditions, can transform ethanol into acetic acid during fermentation [39]. When present in excessive amounts in beer formulations, it imparts a vinegar-like flavor and aroma. The presence of these acids could result from the raw material, wort boiling, or yeast metabolism, or could often have originated from unwanted and uncontrolled microbial contamination [39]. Since beer is not a pasteurized or filtered beverage, a major problem that homebrewers face is beer spoilage. Despite the presence of several inhibitors, such as carbon dioxide, alcohol content (0.5–10% *w*/*w*), and hop compounds, that can increase the microbiological stability of the final product, other microorganisms such as wild yeasts, lactic acid bacteria (LAB), acetic acid bacteria (AAB), *Zymomonas* spp., *Pectinatus* spp., and *Megasphaera* spp., can prevail in the brewing environment, causing problems during the early or late stages of the process [40]. In this study, the produced beers showed low quantities of acetic and lactic acids, and the sensory analysis indicated that they did not entail changes in the acidic flavors, since their flavor threshold is estimated to be 200 mg/L and 400 mg/L, respectively [41].

A sensory analysis showed low differences among samples by the attributes analyzed. However, it is worth noting that the DA was not performed by a trained panel, but by a selected group of brewery experts, which may compromise the sensitivity to variations in the samples. Moreover, this may be due to the low number of crucial VOCs in the beer samples. Indeed, octanol, hexanol, and 2,3-butanediol, which are highly aromatic volatile alcohols with an odor threshold of 1 mg/L, 800 mg/L, and 668 mg/L, respectively [25,27], were present in low amounts in the beer samples. Moreover, considering esters, which bring a fruity amora to fermented beverages, ethyl acetate and ethyl hexanoate levels in the produced beers were below the odor threshold described in the literature (7.5 mg/L and 5.0 mg/L, respectively [25]).

A higher instrumental turbidity was measured in the control than WB beer samples. This can be related to the lower solids content and, thus, release during the maceration process, in dried bread than in wheat flakes [9]. However, differences in turbidity were not observed by the trained panel. Regarding the color attribute, darker colors were observed in the control beer samples (data from EBC and L*-values), in accordance with previous data [9]. This might be putatively due to the baking and drying steps carried out on wasted bread before its addition into the beer formulation. Indeed, these steps could foster Maillard reactions and the production of dark pigments. However, this phenomenon did not change the yellowish color (b*-values); the ΔE parameter showed higher values than the Just Noticeable Difference Values of 2.3 [42], indicating there were noticeable visual differences between beer samples when wheat flakes were substituted by wasted bread. The instrumental data from color measurements and sensory analysis disagreed, in contrast with previous findings [9]. This indicates the importance of both instrumental- and sensory-based data in evaluating foodstuff appearance, along with the further exploration of these contrasting evaluations. Coelho Neto et al. [43], when analyzing the color of Pilsen-style craft beers with adjuncts, observed lighter colors when compared to pure malt beers, and they pointed out that adjuncts can cause a color reduction and are associated with a lower concentration of amino acids and carbohydrates derived from malt in the wort.

Considering beer flavors, the types of adjuncts used can interfere with the flavor profile. It has been previously shown that, in Pilsen-style craft beers, the addition of corn as an adjunct contributes to the increase in the bitterness of beer, while the use of rice as an adjunct has the opposite effect [43]. Meanwhile, Coelho et al. [10] prepared a pale ale beer with stale bread and, in their sensory analysis, found that the participants were not able to detect a noticeable difference in the beer with the addition of bread and the incorporation of stale bread in a 50:50 ratio did not affect the overall character of the beer. A similar result was found in the descriptive analysis of the present study: WB beer did not present off-flavors and aromas, or a marked difference, when replaced by wheat flakes. It is worth noting that the produced WB beers included bread made with wheat flour. This may be the reason why the trained panel did not detect significant differences between the WB and control beers.

In fermented beverages, volatile acids and alcohols are derived from the microbial fermentation of sugars, and, overall, these compounds were identified in a higher concentration in the control beer. However, alcohols can be also formed by the microbial decarboxylation of ketoacids, lipid oxidation, or the degradation of the iso-α-acids of hops during the oxidation of beer [44,45], and acids from lipid hydrolysis or Strecker aldehydes [46]. Volatile acids are important aromatic compounds in fermented beverages, as they can contribute to off-flavors [24,25], and, in the present work, the addition of wasted bread instead of wheat flakes reduced octanoic acid, which presents a sweat/cheesy aroma. On the other hand, volatile alcohols are the most abundant components in beer samples and an important source of flavor [45], and, in the produced beers, could contribute to the beverage aroma bouquet, since the compounds herein were shown to present flower, green, fruit, and malt whiskey aroma.

Aldehydes and ketones are off-flavors in Pilsen, ale, and wheat beers, since they contribute to grass/tallow and herb/butter/resin odors, respectively [25,45]. Acetaldehyde, which contributes to green/apple notes [27], is formed as a metabolic branch point in the pathway from carbohydrate to ethanol and has levels directly related to the ethanol content [47]. Methyl ketones can form through Maillard reactions [48] and may be derived from the drying procedure of barleys to turn them into malt and by the baking and toasting process of WB. These formation pathways are in line with the results from aldehydes and ketones found in the present work.

Esters are critical indicators of quality because they are key compounds for composing the fruity and floral notes of the final product, and, for all esters identified, the addition of wasted bread led to lower amounts of the compounds. Their formation is closely related to fatty acids’ and amino acids’ intracellular metabolism of fermenting yeast cells, and, due to the ethyl esters apolar features, they can diffuse through the cellular membrane into the fermenting medium [49]. Martin-Lobera et al. [9] observed that ale beers were characterized by ethyl octanoate and ethyl decanoate, becoming markers to differentiate it from white bread wasted beers, which were characterized instead by the presence of phenyl ethyl alcohol, in accordance with the present results. Terpenes are usually related to the addition of hops, resulting in a flowery odor in the aroma profile [47], and their profile is in line with our findings. Geraniol and citronellol are known to be influenced by yeast metabolism during fermentation, and sesquiterpenols, esters, and alcohols are only suspected to be bioconverted by yeasts [50]. These results follow the means of the banana and floral aroma intensities in the descriptive analysis. These represent markers aromas of wheat-based beers [51], and, although there were no statistical differences with the control beer, higher average scores were given to beers with wheat flakes only. In industrial production, to maintain the quality and style of beers with wasted bread, they can be marked by the presence of acetaldehyde, which contributes to green/apple notes [27]; this compound is normally one of the most abundant components in the crumb of wheat bread [10,52].

Food waste results in the loss of not only the product itself but also all the resources used in the supply chain [5,53]. The use of wasted bread has proven to be an efficient substitute for wheat flakes. Brancoli et al. [53] investigated the environmental impacts of waste management and ways of valuing surplus bread in Sweden; they found that source reduction has the greatest environmental savings, followed by donation, ethanol production, animal feed, and beer production. These recovery strategies are favored over anaerobic digestion and incineration, which offer the smallest environmental savings. For instance, in beer production, the observed savings of −0.46 kg CO_2_eq in the global warming category are the result of replacing malted barley with surplus bread [53]. Almeida et al. [54] compared the production of standard craft beer with a process that uses surplus bread and found that the latter could reduce environmental impacts by 20%, due to the lower need for barley and the use of grain used as animal feed.

As a limitation, this study included only one type of bread in the beer formulation. Variations in bread ingredients and production methods could influence the results. Additionally, the bread was dried before use, and the utilization of the bakery product without previous treatment can be an alternative, which could save energy and time but might also yield different outcomes. 

Future studies should include toxicological analyses to assess the presence of mycotoxins in the malt, wheat flakes, and bread utilized. Moreover, monitoring changes in *S. cerevisiae* metabolomics and spoilage microorganisms, such as lactic acid bacteria, during the brewing process is crucial in ensuring the quality of the beverage. Further research is needed to thoroughly evaluate the industrial-scale use of wasted bread in beer production.

## 5. Conclusions

Beers produced with the addition of wasted bread demonstrated promising results, indicating that the complete replacement of wheat flakes is a viable and favorable option. During the temperature ramps, the samples with added bread showed some differences in maltose and glucose concentrations, likely influenced by the formulation, processing, and baking of bread, which may have created barriers that hindered starch hydrolysis. In most physico-chemical analyses, the results showed no difference between the control and the beers with the addition of bread; similar results were found in the sensory analysis. For the volatile compounds identified, the beers with the addition of bread stood out for the compounds D-limonene, ethyl dodecanoate, heptanol, acetaldehyde, and ethyl acetate, which should be further explored as volatile markers. On the other hand, in the sensory analysis, several attributes pointed out by the trained panel were equal between the control and the wasted bread beer without wheat flakes. Moreover, no off-flavors were present in the WB beer in comparison to the control. Thus, including wasted bread into beer formulations offers a viable alternative for reusing this food, transforming wasted bread into a new and cost-effective ingredient for brewers.

## Figures and Tables

**Table 1:** Formulations applied for craft beer production: control (no addition of WB), BB1 (addition of 100 g of WB), BB2 (addition of 250 g of WB), and BB3 (addition of 350 g of WB).

Ingredients	Control	BB1	BB2	BB3
Pilsen malt (g)	750	750	750	750
Munich malt (g)	50	50	50	50
Wheat flakes (g)	350	250	100	-
Bread (g)	-	100	250	350
Yeast (mL)	20	20	20	20
Hops (g)	4	4	4	4

**Table 2:** Physico-chemical characteristics of an ale beer produced without (control) and with wasted bread in partial and complete substitution of barley malt.

	Control	BB1	BB2	BB3
pH	4.30 ± 0.02 ^a^	4.07 ± 0.01 ^bc^	4.05 ± 0.0 ^c^	4.13 ± 0.01 ^b^
Acidity (lactic acid (%) *m*/*v*)	0.22 ± 0.70 ^a^	0.22 ± 0.0 ^a^	0.24 ± 0.70 ^a^	0.23 ± 0.0 ^a^
Turbidity (NTU)	102.0 ± 19.8 ^a^	65.4 ± 8.3 ^b^	25.7 ± 6.22 ^c^	54.9 ± 17.3 ^b^
EBC (430 nm)	10.00 ± 0.70 ^a^	7.00± 0.11 ^c^	8.01 ± 0.89 ^b^	7.02 ± 0.34 ^c^
L*	12.2 ± 0.51 ^c^	65.4 ± 8.34 ^a^	25.7 ± 6.22 ^b^	54.85 ± 17.32 ^a^
a*	1.31 ± 0.17 ^a^	0.79 ± 0.06 ^b^	0.605 ± 0.20 ^b^	0.79 ± 0.11 ^b^
b*	10.85 ± 0.47 ^a^	9.84 ± 0.33 ^a^	10.35 ± 0.11 ^a^	10.08 ± 0.56 ^a^
C*	10.93 ± 0.19 ^a^	9.88 ± 0.04 ^a^	10.37 ± 0.13 ^a^	10.11 ± 0.57 ^a^
Δ*E*		53.21	15.32	42.62
Ethanol (g/L)	34.92 ± 3.38 ^a^	28.24 ± 3.16 ^ab^	28.53 ± 0.02 ^ab^	20.21 ± 7.16 ^b^
Glycerol (g/L)	0.81 ± 0.08 ^a^	0.69 ± 0.08 ^a^	0.76 ± 0.0 ^a^	0.65 ± 0.23 ^a^
Acetic acid (g/L)	0.41 ± 0.02 ^a^	0.30 ± 0.05 ^c^	0.38 ± 0.00 ^b^	0.23 ± 0.03 ^d^
Lactic acid (g/L)	1.06 ± 0.16 ^b^	1.09 ± 0.18 ^b^	0.87 ± 0.01 ^b^	1.59 ± 0.74 ^a^
Glucose (g/L)	1.14 ± 0.12 ^a^	1.33 ± 0.16 ^a^	1.75 ± 0.77 ^a^	1.83 ± 0.68 ^a^
Maltose (g/L)	0.00 ± 0.00	1.32 ± 0.18 ^a^	1.15 ± 0.01 ^a^	1.18 ± 0.43 ^a^

^a,b,c,d^ different superscript letters among columns indicate statistical differences at 5% of significance by Tukey’s HSD test.

**Table 3:** Volatile compounds’ (µg/L) profile in ale beer samples (control) and wasted bread beers.

Compounds	Odor	IR _Lit_	IR _Calc_	Control	BB1	BB2	BB3
Acids							
Hexanoic acid ^†^		1847	1846	103.47 ± 12.98 ^a^	66.05 ± 26.27 ^b^	2.88 ± 0.61 ^c^	72.58 ± 16.69 ^b^
5-methylhexanoic acid ^†^		1914	1905	5.10 ± 1.16 ^a^	6.60 ± 0.96 ^a^	2.78 ± 0.15 ^b^	2.82 ± 0.48 ^b^
Heptanoic acid ^††^		1953	1947	13.31 ± 2.00 ^a^	5.51 ± 2.97 ^b^	3.90 ± 0.57 ^b^	7.19 ± 0.86 ^b^
Octanoic Acid ^††^	sweat, cheese	2050	2050	470.87 ± 32.69 ^a^	292.57 ± 13.25 ^b^	249.85 ± 10.48 ^c^	234.52 ± 34.79 ^c^
Alcohols							
Hexanol ^††^	flower, green	1348	1345	2.84 ± 0.06 ^a^	2.69 ± 0.03 ^b^	2.08 ± 0.37 ^c^	2.07 ± 0.12 ^c^
Propanol ^†^	alcohol, pungent	1037	1034	4.12 ± 0.18 ^a^	2.93 ± 0.11 ^b^	2.87 ± 0.39 ^b^	2.98 ± 0.52 ^b^
2-Methyl propanol ^††^		1092	1090	24.22 ± 1.91 ^a^	20.27 ± 0.86 ^a^	19.61 ± 3.00 ^b^	22.48 ± 1.49 ^a^
Butanol ^†^	medicine, fruit	1135	1137	1.69 ± 0.23 ^a^	1.35 ± 0.03 ^b^	1.00 ± 0.36 ^c^	1.09 ± 0.10 ^c^
2-Methyl butanol ^††^	malt	1189	1189	123.76 ± 12.08 ^a^	118.61 ± 0.11 ^a^	97.40 ± 22.76 ^a^	128.07 ± 26.12 ^a^
3-Methyl butanol ^†^	whiskey, malt	1190	1192	531.19 ± 59.91 ^a^	440.36 ± 32.47 ^b^	385.83 ± 48.86 ^b^	479.70 ± 84.11 ^b^
Heptanol ^††^	herb	1447	1447	47.12 ± 5.47 ^b^	53.42 ± 0.84 ^ab^	62.80 ± 4.90 ^a^	35.46 ± 4.46 ^c^
2-Nonanol ^††^	fat, green	1521	1521	5.75 ± 2.08 ^a^	4.05 ± 0.57 ^a^	0.00 ± 0.00	2.63 ± 0.67 ^b^
Octanol ^†^	chemical, metal	1565	1568	9.16 ± 1.53 ^a^	8.41 ± 0.12 ^a^	8.47 ± 1.05 ^a^	8.50 ± 1.17 ^a^
Phenethyl alcohol ^†^		1931	1930	1119.03 ± 15.85 ^a^	782.20 ± 11.37 ^b^	762.74 ± 88.16 ^b^	788.04 ± 110.36 ^b^
3-(Methylthio)-1-propanol ^††^		1719	1719	15.50 ± 9.99 ^a^	8.04 ± 4.26 ^a^	6.24 ± 0.83 ^a^	10.85 ± 2.32 ^a^
2,3-Butanediol ^††^	fruit	1556	1556	5.20 ± 0.57 ^a^	3.82 ± 0.66 ^b^	3.51 ± 1.83 ^ab^	3.42 ± 1.02 ^b^
Aldehyde							
Acetaldehyde ^††^	apple, green	668	748	11.71 ± 0.62 ^a^	5.90 ± 0.26 ^b^	5.52 ± 0.72 ^b^	4.13 ± 0.83 ^b^
Esters							
Ethyl acetate ^†^	pineapple	902	903	145.04 ± 34.95 ^a^	80.30 ± 0.45 ^b^	88.85 ± 5.42 ^b^	70.17 ± 14.54 ^b^
2-Methylpropyl acetate ^††^		1005	1003	3.31 ± 1.01 ^a^	1.46 ± 0.12 ^b^	1.77 ± 0.61 ^b^	1.82 ± 0.99 ^b^
Ethyl butanoate ^†^		1025	1022	7.77 ± 2.91 ^a^	4.85 ± 0.32 ^a^	5.18 ± 0.70 ^a^	3.72 ± 0.06 ^b^
3-Methylbutyl acetate ^††^		1105	1106	345.77 ± 136.15 ^a^	131.67 ± 5.63 ^bc^	155.14 ± 16.28 ^b^	109.74 ± 34.21 ^c^
2-Methylbutyl isobutyrate ^††^		1185	1173	2.58 ± 1.46 ^a^	2.46 ± 0.04 ^a^	0.24 ± 0.07 ^b^	0.00 ± 0.00
Ethyl hexanoate ^†^	fruit, jelly palm	1205	1206	251.00 ± 30.38 ^a^	153.18 ± 0.46 ^b^	151.34 ± 29.30 ^b^	150.46 ± 39.39 ^b^
Hexyl acetate ^††^	fruit, herb	1255	1254	3.17 ± 0.17 ^a^	1.71 ± 0.21 ^b^	1.13 ± 0.55 ^b^	0.96 ± 0.70 ^b^
Ethyl heptanoate ^††^		1317	1318	51.95 ± 26.13 ^a^	47.93 ± 3.37 ^a^	32.71 ± 5.58 ^a^	17.91 ± 20.08 ^a^
Heptyl acetate ^††^		1360	1358	41.54 ± 20.85 ^a^	30.26 ± 0.50 ^a^	29.38 ± 5.45 ^a^	10.54 ± 2.75 ^b^
Ethyl hexenoate ^††^		1360	1370	29.83 ± 3.86 ^a^	13.80 ± 1.49 ^b^	13.29 ± 2.40 ^b^	16.55 ± 7.07 ^b^
Ethyl octanoate ^†^	fruit	1420	1421	3383.87 ± 459.43 ^a^	1850.55 ± 211.80 ^b^	1578.20 ± 184.16 ^b^	1467.25 ± 489.29 ^b^
2-Methylbutyl hexanoate ^††^		1451	1453	3.22 ± 1.38 ^a^	2.76 ± 1.05 ^a^	2.86 ± 0.27 ^a^	2.46 ± 0.83 ^a^
Ethyl nonanoate ^††^		1531	1534	14.66 ± 6.57 ^a^	14.31 ± 2.65 ^a^	8.99 ± 0.54 ^b^	12.44 ± 7.00 ^ab^
Ethyl 8-nonenoate ^††^		1581	1594	2.99 ± 1.41 ^a^	2.70 ± 2.43 ^a^	1.01 ± 0.20 ^a^	1.62 ± 0.42 ^a^
Ethyl decanoate ^††^		1624	1626	292.12 ± 42.47 ^a^	241.64 ± 60.52 ^a^	112.77 ± 16.61 ^b^	128.69 ± 71.41 ^b^
3-Methylbutyl octanoate ^††^		1648	1648	22.00 ± 5.61 ^a^	30.28 ± 11.22 ^a^	11.99 ± 6.29 ^b^	24.75 ± 24.07 ^ab^
Ethyl E-4-decenoate ^††^		1680	1650	9.36 ± 5.08	0.00 ± 0.00	0.00 ± 0.00	0.00 ± 0.00
Ethyl E-9-decenoate ^††^		1685	1680	808.59 ± 401.10 ^a^	790.25 ± 142.63 ^a^	468.50 ± 14.16 ^a^	512.60 ± 269.00 ^a^
2-Phenylethyl acetate ^††^		1822	1823	466.59 ± 2.31 ^a^	169.08 ± 10.83 ^b^	146.82 ± 18.42 ^b^	126.75 ± 14.14 ^b^
Ethyl dodecanoate ^††^		1846	1845	0.00 ± 0.00	54.92 ± 8.69 ^a^	67.64 ± 7.93 ^a^	0.00 ± 0.00
Ethyl benzenepropanoate ^††^		1892	1892	6.75 ± 2.18 ^a^	1.67 ± 0.64 ^b^	1.38 ± 0.78 ^b^	0.79 ± 0.27 ^b^
Ketones							
3-Methyl-5-heptanone ^††^		1219	1238	7.91 ± 4.45 ^b^	5.57 ± 0.57 ^b^	21.22 ± 0.80 ^a^	8.37 ± 3.03 ^b^
6-Methyl-5-hepten-2-one ^††^		1322	1325	1.57 ± 0.15 ^a^	0.99 0.01 ^b^	0.77 ± 0.04 ^b^	0.55 ± 0.78 ^b^
Terpenes							
D-Limonene ^††^	lemon, orange	1160	1162	1.18 ± 0.37 ^c^	2.57 ± 0.28 ^a^	1.51 ± 0.74 ^bc^	1.71 ± 0.09 ^b^
β-Linalool ^†^	flower, lavender	1552	1553	43.58 ± 2.57 ^a^	27.27 ± 2.49 ^b^	12.70 ± 3.20 ^d^	22.33 ± 2.88 ^c^
β-Citronellol ^††^	rose	1765	1765	11.03 ± 2.01 ^a^	11.52 ± 5.01 ^b^	6.95 ± 0.18 ^c^	9.02 ± 0.59 ^b^
Z-Geraniol ^†^	rose	1861	1859	4.69 ± 0.31 ^a^	4.02 ± 0.74 ^a^	2.34 ± 0.37 ^b^	2.82 ± 0.14 ^b^
Polycyclic Aromatic Hydrocarbon ^††^							
2-Methyl naphthalene ^††^		1863	1863	4.90 ± 1.98 ^a^	3.69 ± 0.26 ^a^	2.24 ± 0.25 ^b^	3.70 ± 1.22 ^ab^

^a,b,c,d^ different superscript letters in the same line indicate significant differences among samples (*p* < 0.05) by Tukey’s HSD test. ^†^ positively identified by comparing the retention times and spectra of standard compounds. ^††^ identified by comparing the experimental mass spectrum with those found in the NIST 14 spectra library.

## Data Availability

The data presented in this study are available on request from the corresponding author. Data are not publicly available due to ethical restrictions.

## Supplementary Materials

The following supporting information can be downloaded at: https://www.mdpi.com/article/10.3390/microorganisms13010066/s1, Table S1: Attributes’ description used in the descriptive analysis of beers.
