# Supplementary material for: Understanding the Utilization of Wasted Bread as a Brewing Adjunct for Producing a Sustainable Wheat Craft Beer
Source: Microorganisms. 2025 Jan 2;13(1):66. doi: 10.3390/microorganisms13010066 (PMC11767916; doi:10.3390/microorganisms13010066)
Supplement: Supplementary file 1 [file microorganisms-13-00066-s001.zip › microorganisms-3264342-supplementary.pdf]

Understanding the Utilization of Wasted Bread as a Brewing Adjunct for Producing a Sustainable Wheat Craft Beer

Katry Dall'Acua, Manuela Poletto Klein, Bárbara Iegli Tech, Alessandra Fontana, Ludmylla Tamara Crepalde, Roger Wagner, Fernanda de Candido de Oliveira and Voltaire Sant'Anna

Supplementary material

Table S1. Attributes' description used in the descriptive analysis of beers.

Category	Attributes	Description
Appearance	Yellow color	Golden yellow color of the beverage
	Dark color	Black color of the beverage
	Turbidity	Foggy/turve appearance of the beverage
	Foaming	Foam that persists for at least 15 seconds after the beverage is served into the cup
Aroma	Banana	Scent of banana
	Clove	Scent of clove
	Malt	Scent of barley malt
	Floral	Flowery scent
Taste/Flavor	Banana	Banana flavor
	Clove	Clove flavor
	Malt	Barley malt flavor
	Alcoholic	Flavor of alcohol
	Acidity	Taste of acid
	Sweetness	Sweet taste
	Refreshment	Mouthfeel of refreshment
	Bitterness	Bitter taste
	Sparkling	Carbon dioxide bubbles felt in the mouth

After taste	Residual taste	Taste that remains in the mouth after 10 seconds
	Persistent taste	Taste that remains in the mouth after 20 seconds
